# Hemodynamic phenotype-based, capillary refill time-targeted resuscitation in early septic shock: The ANDROMEDA-SHOCK-2 Randomized Clinical Trial study protocol

**DOI:** 10.5935/0103-507X.20220004-en

**Published:** 2022

**Authors:** Eduardo Kattan, Jan Bakker, Elisa Estenssoro, Gustavo Adolfo Ospina-Tascón, Alexandre Biasi Cavalcanti, Daniel De Backer, Antoine Vieillard-Baron, Jean-Louis Teboul, Ricardo Castro, Glenn Hernández

**Affiliations:** 1 Departamento de Medicina Intensiva, Facultad de Medicina, Pontificia Universidad Católica de Chile - Santiago, Chile.; 2 Department of Pulmonology and Critical Care, Columbia University Medical Center - New York, United States.; 3 Servicio de Terapia Intensiva, Hospital Interzonal de Agudos San Martin de La Plata - Buenos Aires, Argentina.; 4 Department of Intensive Care Medicine, Universidad ICESI, Fundación Valle del Lili - Cali, Colombia.; 5 Research Institute, HCor-Hospital do Coração - São Paulo (SP), Brazil.; 6 Department of Intensive Care, CHIREC Hospitals, Université Libre de Bruxelles - Brussels, Belgium.; 7 Intensive Care Medicine Unit, Assistance Publique-Hôpitaux de Paris, University Hospital Ambroise Paré - Boulogne-Billancourt, France.; 8 Service de Médecine Intensive-Réanimation, Hôpital de Bicêtre, Université Paris-Saclay - Le Kremlin-Bicêtre, France.

**Keywords:** Sepsis, Septic shock, Capillary refill time, Phenotype, Critical care, Length of stay, Perfusion, Norepinephrine, Echocardiography, Algorithm

## Abstract

**Background:**

Early reversion of sepsis-induced tissue hypoperfusion is essential for survival in septic shock. However, consensus regarding the best initial resuscitation strategy is lacking given that interventions designed for the entire population with septic shock might produce unnecessary fluid administration. This article reports the rationale, study design and analysis plan of the ANDROMEDA-2 study, which aims to determine whether a peripheral perfusion-guided strategy consisting of capillary refill time-targeted resuscitation based on clinical and hemodynamic phenotypes is associated with a decrease in a composite outcome of mortality, time to organ support cessation, and hospital length of stay compared to standard care in patients with early (< 4 hours of diagnosis) septic shock.

**Methods:**

The ANDROMEDA-2 study is a multicenter, multinational randomized controlled trial. In the intervention group, capillary refill time will be measured hourly for 6 hours. If abnormal, patients will enter an algorithm starting with pulse pressure assessment. Patients with pulse pressure less than 40mmHg will be tested for fluid responsiveness and receive fluids accordingly. In patients with pulse pressure > 40mmHg, norepinephrine will be titrated to maintain diastolic arterial pressure > 50mmHg. Patients who fail to normalize capillary refill time after the previous steps will be subjected to critical care echocardiography for cardiac dysfunction evaluation and subsequent management. Finally, vasopressor and inodilator tests will be performed to further optimize perfusion. A sample size of 1,500 patients will provide 88% power to demonstrate superiority of the capillary refill time-targeted strategy.

**Conclusions:**

If hemodynamic phenotype-based, capillary refill time-targeted resuscitation demonstrates to be a superior strategy, care processes in septic shock resuscitation can be optimized with bedside tools.

## INTRODUCTION

Early recognition and prompt reversion of sepsis-induced tissue hypoperfusion are key factors in determining the survival of patient experiencing septic shock.^(1,2)^ Notwithstanding extensive research, mortality due to septic shock remains substantially high, and there is no consensus about the best initial resuscitation strategy. Indeed, resuscitative interventions assuming the principle of *one size fits all* run the risk of unnecessary fluid administration and harmful volume accumulation.^(3)^

Recent evidence suggests that peripheral perfusionguided strategies during the early stages of septic shock might be associated with lower mortality, faster recovery of organ dysfunction and reduced intensity of therapeutic interventions compared with lactate-guided resuscitation.^(4,5)^ In fact, guiding resuscitation utilizing evaluation of peripheral perfusion with capillary refill time (CRT) resulted in a significant reduction in administered fluids and vasoactive-related interventions.^(6)^ This resuscitation strategy could also limit unnecessary therapeutic interventions performed in the presence of persistently high lactate levels.^(6)^ Thus, CRT has been proposed to guide resuscitation in the intensive care unit (ICU) and, potentially, in pre-ICU or resource- limited settings.^(2,7)^

Mechanisms involved in circulatory failure are complicated and frequently overlap in septic shock. Consequently, resuscitative interventions should be integrative and individualized according to particular macrohemodynamic features detected during clinical assessment.^(8)^ For example, some patients might remain hypovolemic even after initial fluid loading, which would suggest a potential benefit from additional volume administration.^(9)^ Others exhibiting very low diastolic arterial pressures (DAPs) could benefit from increasing the vasopressor dose instead of administering additional and potentially harmful fluid loading.^(10-12)^ Similarly, early identification of left/right ventricular dysfunction could lead to early hemodynamic adjustment to limit or avoid deleterious interventions.^(13)^ Therefore, a characterization of these cardiovascular phenotypes in septic shock might lead to more personalized resuscitation and improved outcomes. Despite extensive research, a universal method for the identification of such phenotypes has not yet been agreed upon.^(13-15)^

In an effort to characterize clinical and hemodynamic phenotypes in patients with early septic shock and thus target the most adequate therapeutic approaches, the ANDROMEDA-SHOCK-2 (A2) study will integrate different variables, such as the systematic assessment of fluid responsiveness, pulse pressure as a surrogate of stroke volume, DAP for evaluation of vascular tone, and selective echocardiography for assessment of myocardial dysfunction.^(10,13,16-18)^

We hypothesize that CRT-targeted resuscitation based on clinical hemodynamic phenotyping in patients with septic shock will improve a composite hierarchical outcome of mortality, time to cessation of vital support, and length of hospital stay within 28 days compared with standard care.

## METHODS

### Main objective

To determine whether CRT-targeted resuscitation based on clinical hemodynamic phenotyping is associated with a decrease in a hierarchical composite outcome within 28 days after randomization, which includes mortality, time to cessation of vital support, and length of hospital stay, compared to standard care in patients with early septic shock.

### Secondary objective

To determine whether CRT-targeted resuscitation based on clinical hemodynamic phenotyping is associated with a decrease in all-cause mortality within 28 days after randomization, more organ support-free days within 28 days after randomization, and a decreased length of hospital stay within 28 days after randomization compared to standard care in patients with early septic shock.

### Primary outcome

The primary outcome is a hierarchical composite of all-cause mortality, time to cessation of vital support (truncated at 28 days) and length of hospital stay within 28 days after randomization.

### Secondary outcomes

Secondary outcomes are: all-cause mortality within 28 days after randomization; organ support-free days within 28 days; and length of hospital stay (truncated at 28 days).

### Other (tertiary) clinical outcomes

Other clinical outcomes are: all-cause mortality within 90 days after randomization; length of hospital stay; length of ICU stay; time to cessation of vasopressor support; time to cessation of mechanical ventilation (MV); time to cessation of renal replacement therapy (RRT); vasopressor support-free days; MV-free days; RRT-free days; variation in Sequential Organ Failure Assessment (SOFA) score;^(19)^ variation in creatinine-based Kidney Disease: Improving Global Outcomes (KDIGO) stage;^(20)^ volume of resuscitation fluids; net fluid balance; Evolution of CRT; evolution of lactate levels; evolution of central venous pressure (CVP); evolution of central venous oxygen saturation (ScvO2); and evolution of the central venous to arterial carbon dioxide difference (delta pCO2(v-a))

### Study design

ANDROMEDA-SHOCK-2 will be a multicenter, open-label, investigator-generated, randomized controlled trial conducted under supervision of an independent Data Safety Monitoring Board (DSMB). Recruited patients will be randomized to CRT-P or to standard care.

### Patients

#### Inclusion criteria

Consecutive adult patients (≥ 18 years) with septic shock according to the Sepsis-3 consensus conference will be considered eligible. Septic shock is defined as suspected or confirmed infection and norepinephrine requirements due to persistent hypotension after a fluid load of at least 1000mL in 1 hour, plus the presence of hyperlactatemia (> 2mmol/L).^(21)^

#### Patients will be excluded based on the following criteria

We will exclude patients with: more than 4 hours since diagnosis of septic shock anticipated surgery or acute hemodialysis procedure to start during the 6-hour intervention period; active bleeding; do-not-resuscitate status; Child B-C cirrhosis; underlying disease process with a life expectancy < 90 days and/or the attending clinician deems aggressive resuscitation unsuitable; pregnancy; concomitant severe acute respiratory distress syndrome (ARDS); and patients in whom CRT cannot be accurately assessed.

Screening will be conducted in the ICU. Clinical investigators at each participating center will be responsible for screening all patients who fulfil the inclusion criteria. A screening log will be generated to register all patients with septic shock regardless of whether they are eligible for study inclusion.

### Randomization and blinding

A randomization sequence with a 1:1 allocation will be generated using a computer program and captured using an electronic data management system. Study group assignment will be performed by means of randomized permuted blocks of variable size. Allocation concealment will be maintained by means of central randomization.

Given that the intervention will be administered to critically ill patients (mostly sedated), blinding of these patients is not necessary. Because this is a nonpharmacological intervention, blinding the medical team is not feasible.

### Interventions

#### General management for both groups

Sepsis source identification and treatment should be pursued as a priority of first-line treatment. A central venous catheter and an arterial line will be inserted in all cases. The use of a pulmonary artery catheter or a pulsecontour continuous cardiac output device is not part of the CRT-P protocol but may be used in conditions in which attending physicians consider it for safety reasons.

Norepinephrine will be the vasopressor of choice and will be adjusted to achieve and maintain a mean arterial pressure (MAP) ≥ 65mmHg in all patients. Hemoglobin concentrations will be maintained at 7 - 8g/ dL or greater to optimize arterial O2 content. Stress ulcer prophylaxis, glycemic control, prophylaxis of deep venous thrombosis, and MV settings will be managed according to current recommendations.^(2)^ Rescue therapies, such as epinephrine, vasopressin analogs, and steroids, or different blood purification techniques, such as high-volume hemofiltration, in evolving patients will be administered according to the standard practice of each center.

### Study protocol

**Figure 1:**
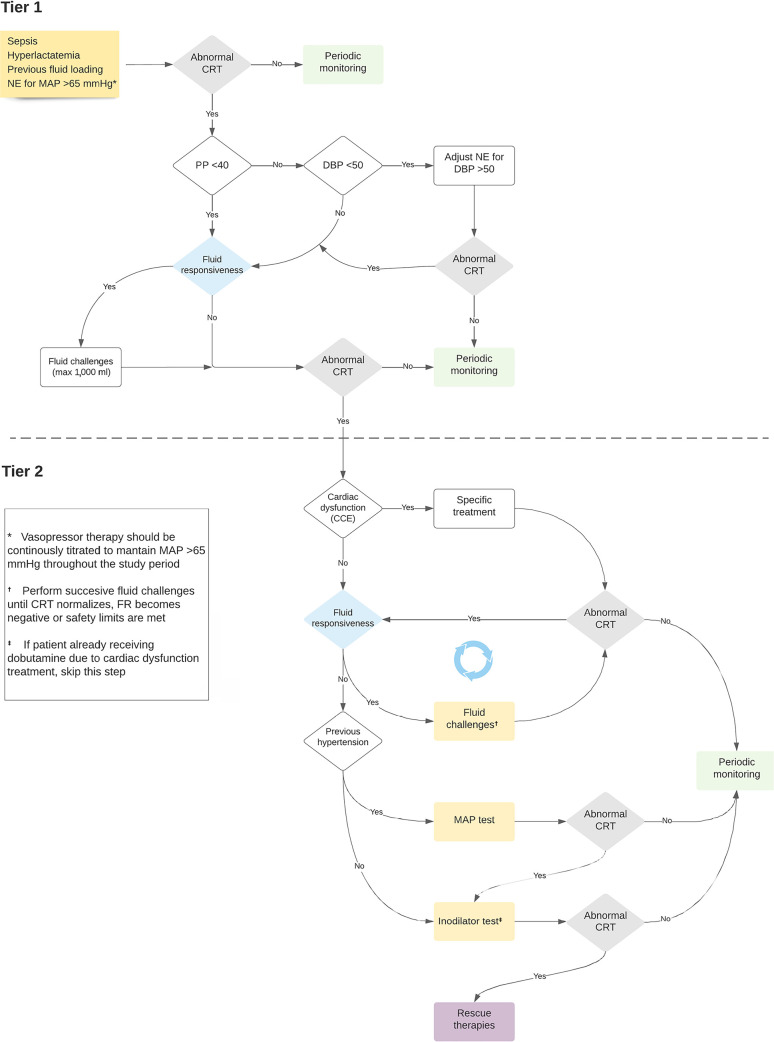
Study protocol flow diagram. NE - norepinephrine; MAP - mean arterial pressure; CRT - capillary refill time; PP - pulse pressure; DBP - diastolic blood pressure; CCE - critical care echocardiography; FR - fluid responsiveness.

A sequential approach to resuscitation will be followed in the CRT-P group, as shown in figure 1. Time 0 is the starting point after randomization when a central venous catheter and an arterial line are already in place, and the basal measurements are performed, including hemodynamics and blood sampling. The study period will last 6 hours. Thereafter, attending physicians may continue to treat patients according to their standard practice or department protocol.

### Tests and procedures during the study period

#### Intervention group (CRT-P)

These patients should follow a management algorithm as described in figure 1.

Patients with normal CRT at baseline will proceed to periodic monitoring (every hour/6 hours) and start the algorithm if CRT becomes abnormal at any of these timepoints. Patients with abnormal CRT will follow the loop when fulfilling the Sepsis-3 definition of septic shock. The first categorization will be performed according to pulse pressure.^(16,17,22)^ Patients with a pulse pressure < 40mmHg, will undergo fluid responsiveness assessment.

Fluid responsiveness assessments will be performed using the technique preferred by each center, but supplementary recommendations with technical details will be provided in the Manual of Operations.^(9,23-26)^ Passive-leg raising with pulse pressure may be acceptable in spontaneous breathing patients. However, considering the possibility of false negatives, it is not the preferred technique.^(9,26,27)^

Fluid responsiveness-negative patients or those with undetermined status should proceed to obligatory critical care echocardiography (CCE) to rule out significant cardiac dysfunction (Figure 1) and proceed accordingly.^(28,29)^ Fluid boluses (500mL of crystalloids or 5% albumin) will be administered in 30-minute intervals with a maximum of 1000cc (2 fluid challenges) provided that the patient remains fluid responsiveness-positive and no safety issues appear (increase in CVP > 5mmHg or other congestion signals). Patients with safety signals should proceed immediately to CCE (Figure 1).

Patients with pulse pressure ≥ 40mmHg will follow the right arm of the algorithm (Figure 1) will proceed according to DAP. If DAP is ≥ 50mmHg, then the patient will move to fluid responsiveness assessment.^(10,30,31)^ If DAP is < 50mmHg, then norepinephrine will be increased to achieve a MAP > 65mmHg and a DAP ≥ 50 mmHg. Capillary refill time will be assessed 1 hour later. Norepinephrine will be increased in 0.1mics/kg/minute increments up to 0.5mcg/kg/minute. When reaching 0.5mcg/kg/minute, only a 25% additional increment will be acceptable if the DAP goal still has not been reached (max 0.625mcg/kg/minute). Norepinephrine may be stopped earlier if potential adverse effects are observed (heart rate - HR) > 130 bpm, arrhythmias, or evident cardiac ischemia). In centers in which vasopressin is part of the standard of care, it may be added when norepinephrine > 0.3mics/kg/minute, but up to a limit of 0.04U/minute to obtain a DAP ≥ 50mmHg.

If the CRT is normal, patients will proceed to periodic monitoring. Patients with persistent abnormal CRT or that has reached norepinephrine or vasopressin safety limit will proceed directly to CCE according to the results. Patients who correct CRT with first-tier interventions (fluid boluses on one side and norepinephrine adjustment in the other) will not be subjected to obligatory CCE but will simply proceed to periodic monitoring.

When a patient fails to correct CRT after the whole algorithm procedure, rescue therapies should be considered by attending physicians. These therapies will not be standardized since the protocol is interrupted at that point; however, monitoring and registration during the 6 hours intervention period and thereafter continues until the end of the study.

### Cardiac dysfunction definitions

- **Left ventricular (LV) systolic dysfunction** is the association of a LV fractional area change less than 40% plus an aortic velocity time integral (VTI) less than 14.^(13,28)^
- **Right ventricular (RV) failure** is the association of RV dilatation (RV/LV area > 1 plus a CVP at least greater than 8mmHg).^(13,28)^

### Cardiac dysfunction suggested management

- In the case of LV systolic dysfunction, a low dose of dobutamine may be considered, starting with 2.5mcg/kg/minute and up to 7.5mcg/kg/minute. Dobutamine should be stopped if HR increases > 20% over 120 bpm or arrhythmias, ischemia or hypotension develop.^(13,28)^
- In the case of RV failure, MV settings should be adjusted to decrease PEEP less than 10cmH2O and limit plateau pressure below 28cmH2O. If patients develop severe ARDS, the prone position should be considered. From a hemodynamic point of view, no further fluid administration is recommended.^(13,28)^

### Additional fluid challenges

In patients with persistent abnormal CRT and for whom cardiac dysfunction is excluded by CCE, further fluid responsiveness assessment is warranted. If patients present fluid responsiveness + status, additional fluid challenges will be administered following the same safety rules as mentioned above.

#### Vasopressor test

In patients with a previous history of chronic hypertension and persistently abnormal CRT after following all previous resuscitation steps, an open-label vasopressor test will be performed, increasing MAP up to 80 - 85mmHg using progressive incremental doses of norepinephrine. Parameters will be reassessed after 1 hour. If CRT improves, norepinephrine will be titrated to maintain this new MAP goal throughout the study period. If goals are not achieved despite increasing MAP or adverse effects are observed (HR > 130 bpm, arrhythmias, or evident cardiac ischemia), the norepinephrine dose will be reduced to the level before the vasopressor test, and the protocol will move to the next step.(4,32,33)

#### Inodilator test

An open-label test of dobutamine at a fixed 5mcg/kg/ minute dose (at the discretion of the attending physician) will be started in nonchronic hypertensive patients with persistent abnormal CRT and negative fluid responsiveness status. Capillary refill time will be controlled after 1 hour, as noted in the vasopressor test. If still abnormal, dobutamine will be discontinued, and no further action will be taken during the study period. Dobutamine will be maintained throughout the study period in those favorably responding to the open label inodilator test. As a safety measure, inodilators will be stopped if HR increases > 20% over 120 bpm or arrhythmias, ischemia or hypotension develop.^(4)^ Patients in whom dobutamine was started earlier due to LV dysfunction should not be subjected to the inodilator test.^(4,32,33)^

### Capillary refill time assessment

Capillary refill time will be measured by applying firm pressure to the ventral surface of the right index finger distal phalanx with a glass microscope slide. The pressure will be increased until the skin is blank and then maintained for 10 seconds. The time for return of the normal skin color will be registered with a chronometer. A CRT > 3 seconds will be considered abnormal.^(4,32,33)^

### Standard care group

Patients allocated to the standard care group will be managed by the clinical staff according to standard practice at their sites, including decisions about hemodynamic and perfusion monitoring and all treatments, but should follow general recommendations of the Surviving Sepsis Campaign to avoid extremes of clinical practice.^(2)^ These recommendations include basic hemodynamic targets, such as a MAP > 65mmHg, HR < 120bpm, oxygen saturation (SatO_2_) > 94%, hemoglobin (Hb) > 7 - 8gr/dL, and the use of norepinephrine as the first vasopressor and crystalloids as the fluid of choice. All data regarding the insertion of invasive monitoring devices, intravenous-fluid resuscitation, vasoactive support, MV, and other supportive therapies will be collected by the study coordinator or monitors. Leading investigators at a site will not serve as the bedside treating physician for patients in the standard care group.

### Safety measures

The protocol can be stopped at any moment for safety considerations during the 6-hour study period if the attending intensivist considers that the patient has developed unexpected and severe complications or conditions that under his judgment require liberalization of management. This action must be reported on the case report form, and the patient will be followed up with major outcomes and included in the intention-to-treat analysis. Specific safety measures for fluid administration, vasopressor and inodilator tests are provided above and in the Manual of Operations.

In addition, even if resuscitation is stopped after achieving the CRT target in the CRT-P group, further hemodynamic interventions may be decided by attendings for interpreting and addressing unstable or severely abnormal circulatory variables, such as HR > 130 bpm, MAP less than 60mmHg with norepinephrine > 0.5mcg/ kg/minute, among others.

### Suspected unexpected serious adverse reaction

Any adverse event that occurs in a clinical trial subject and is assessed by the study investigator as being unexpected, serious and as having a reasonable possibility of a causal relationship with the study procedure will be reported. Reports of these reactions are subject to expedited submission to health authorities. Suspected unexpected serious adverse reactions will be analyzed by the SCC and DSMB.

### Data collection and management

Study follow-up and the variables that will be collected are described in table 1.

**Table 1:** Relevant variables to be registered during the study period

Baseline
Demographics, comorbidities, APACHE-II score, SOFA score
Sepsis source, treatment, adequacy of treatment, time from shock initiation to first antibiotics
Pre-ICU resuscitation administered fluid and fluid balance, AKI-KDIGO criteria^(19,20)^
Hemodynamics: HR, SAP, MAP, DAP, CVP, NE dose
Perfusion variables: lactate, ScvO2, delta pCO2(v-a), hemoglobin, CRT, mottling score
**Evolution**
SOFA score at 8, 24, 48 and 72 hours and at 4, 5 and 7 days
AKI criteria at 8, 24, 48 and 72 hours
Hemodynamics hourly up to 6 hours
Fluid administration and balance at 6, 24, 48 and 72 hours
Complete perfusion assessment at 6, 24, 48 and 72 hours
Register of vasoactive drugs and dobutamine/milrinone use
Register of CCE
Register of FR status and techniques
Register of MV and RRT techniques
Adjuvant therapies: high-volume hemofiltration, use of vasopressin, epinephrine, others
Follow-up until 28 days for use of MV, RRT and vasopressors
All-cause mortality at hospital discharge and at 28 and 90 days
Cause of death

APACHE II - Acute Physiology And Chronic Health Evaluation II; SOFA - Sequential organ failure Assessment; ICU - intensive care unit; AKI - acute kidney injury; KDIGO - Kidney Disease: Improving Global Outcomes; HR - heart rate; SAP - systolic arterial pressure; MAP - mean arterial pressure; DAP - diastolic arterial pressure; CVP - central venous pressure; NE - norepinephrine; ScvO2 - central venous oxygen saturation; delta pCO2(v-a): difference between central venous carbon dioxide pressure and arterial carbon dioxide pressure; CRT - capillary refill time; CCE - critical care echocardiography; FR - fluid responsiveness; MV - mechanical ventilation; RRT - renal replacement therapy.

### Data handling and record keeping

Individual patient data will be handled as ordinary chart records and will be kept according to the legislation (e.g., data protection agencies) of each participating country. Data will be directly registered in the electronic case report form which is Health Insurance Portability and Accountability Act (HIPAA) compliant. Data will be captured and stored at Erasmus Medical Center for further quality control and statistical analysis.

All original records (including consent forms, case report forms, serious adverse event reports, and relevant correspondence) will be retained at trial sites to allow inspection by relevant authorities. The trial database will be maintained for and anonymized if requested for revision.

### Quality control

Several procedures will assure data quality, including online study protocol and electronic form registry training and availability from the SCC to resolve issues or problems that may arise. A data quality team (DQT) will ensure the adequacy of the online registry, identify missing values and inconsistencies, and contact study centers and SSC to promptly solve these issues.

### Missing data management

Missing data will be reported in the publication. If further analyses reveal substantial missingness, multiple imputation will be considered.

### Sample size

We assumed a mortality of 39% in the control group, which is based on the mortality of the whole cohort in ANDROMEDA-SHOCK.^(4)^ Given that the intervention arm includes several new steps aimed at better tailoring fluid administration, we expect a further decrease in the use of fluids. Based on ANDROMEDA-SHOCK’s data, this new algorithm based on pulse pressure, diastolic blood pressure, and echocardiography could reduce fluid administration in at least 60% of these patients. Previous studies have shown that a reduction in fluid administration is associated with better outcomes. Thus, we estimate a 6% reduction in mortality. Estimates of the number of days needing life support truncated at 28 days and length of hospital stay in the control group were based on data from the ANDROMEDA-SHOCK control group.

For the control group, we assumed a 28-day mortality of 39%, a number of days needing life support truncated in 28 days of 5.6 (standard deviation - SD, 6.7) and a length-of-hospital stay truncated in 28 days of 15.6 days (SD, 10.1). We considered that the experimental group treatment would reduce mortality to 33% (absolute reduction of 6%), shorten days using life support to 4.3 (SD, 6.2) and shorten length of hospital stay to 14 days (SD, 9.9). Length-of-hospital and number of days needing life support were simulated assuming a beta-binomial distribution within 28 days using parameters acquired in the ANDROMEDA-SHOCK trial.^(4)^

With 1,500 patients enrolled, the trial will have a power of 88% to show superiority in the win-ratio outcome for a two-sided α of 0.05.

### Statistical analysis

A detailed statistical analysis plan will be elaborated and published before the trial database is locked and data are analyzed. The fundamental characteristics of the statistical analysis plan are described below.

All patients will be analyzed in the groups they were assigned to independently of adherence to trial protocol, i.e., analysis will follow the intention-to-treat principle. For patients with missing data for the primary outcome, we will impute missing primary outcome data using multiple imputation chains. Variables to be used for imputation will be specified in the statistical analysis plan and will likely include age, enrolling site, and Acute Physiology and Chronic Health Evaluation (APACHE) II score. The medians of the imputed results (or the most frequent category) will be used for the analysis. Categorical variables will be presented as numbers and percentages. Continuous variables will be presented as the mean and SD or median and interquartile range if the distribution is asymmetrical.

We will assess the effect of the trial treatment compared to the control group treatment on the hierarchical primary outcome using the win ratio method proposed by Dong et al.,^(34)^ which is based on the Pocock et al. win ratio,^(35)^ using treatment as a fixed effect. All patients in the treatment group will be compared to all patients in the control group, one pair at a time, following the hierarchical primary outcome: all-cause mortality within 28 days, time to cessation of vital support within 28 days, and length of hospital stay (truncated at Day 28).

Thus, in each pair, mortality will be compared. If only one of the patients died, a win will be counted for the group of surviving patients. If both patients in the pair died, this will be considered a tie (and no further comparisons will be made for this pair). If both patients survive past 28 days, then the time to cessation of vital support will be compared. A win will be counted for the treatment group, which has the patient with the shortest time to cessation of vital support. If the number of days up to cessation of vital support is the same within the pair, then the third outcome in the hierarchy, namely, length of hospital stay, will be compared. A win will be counted for the treatment group that has the patient with the shortest length of hospital stay. Of the length of stay (in number of days) is identical, then this will be counted as a tie.

The win ratio is calculated as the total number of wins in the experimental group divided by the total number of wins in the control group. A value greater than 1 indicates better outcomes in the experimental group. The bootstrap resampling method will be used to calculate the 95% confidence interval (95%CI) for the win ratio and P value for the hypothesis test.

We will assess the treatment effect on the primary hierarchical composite outcome according to the following subgroups: age (< or ≥ 65 years); APACHE II (< or ≥ 25 points); SOFA (< or ≥ 10 points); baseline lactate (< or ≥ 4mmol/L); baseline CRT (< or ≥ 3 seconds); septic shock source (lung *versus* abdominal *versus* urinary *versus* soft tissues *versus* bacteremia *versus* other); and MV at inclusion.

P values will be reported for the primary outcome analysis, and p values for interaction will be reported for the subgroup analyses; the remaining outcomes will be reported with the mean effect and 95%CI. Given the potential for type I error due to multiple comparisons, the findings for the analyses of the secondary and tertiary outcomes should be interpreted as exploratory. All tests are 2-sided with an α level of 0.05. All analyses will be performed with the latest version of R software (R Foundation for Statistical Computing).

### Ethics and informed consent

Each investigator center will submit the study protocol to its Institutional Review Board (IRB). The study will start only after being approved by the IRB. Written informed consent will be obtained from a legal representative of all participants. This study follows local and international declarations. The informed consent form will be translated to all involved languages (countries).

### Trial organization and management

**Steering Committee:** the Steering Committee is responsible for the overall study supervision, assisting in developing the study protocol and preparing the final manuscript. All other study committees report to this committee. Its members are investigators trained in designing and conducting randomized clinical trials in critically ill patients.

**Advisory Board:** the Advisory Board is a committee of experts in the field that will advise the Steering Committee on different requested aspects and will also promote and organize substudies.

**Study Coordinating Center:** this Committee is the executive committee, conducting the trial in all organizational, logistic, and procedural aspects as well as controlling the DQT.

**Data Safety Monitoring Board:** the DSMB comprises independent epidemiologists and intensivists who supervise the trial.

Interim analyses will be conducted after recruitment of the first 200 patients and at 75% of the sample by the DSMB. The first interim analysis will have two steps:

- A safety analysis where 28-day mortality will be compared between anonymized groups masked as A or B to detect potential risks associated with the intervention. According to the results, the DSMB may recommend the interruption of the study for safety reasons.
- A second blinded-to-outcome analysis will be performed to compare the use of selected procedural interventions such as resuscitation fluids during the 6-hour study period between groups to demonstrate a gradient. If no difference or gradient is found, the DSMB may recommend interruption of the study for futility reasons.

The second interim analysis will focus on differences in the primary outcome between groups to potentially recommend an earlier interruption of the study in case of an already statistically significant difference.

### Study Centers

Approximately one hundred centers will be recruited in Western Europe, Asia, North America, and Latin America. The process will start with a survey of professional and technical resources as well as processes of care. Centers will be contacted to make this process representative across public, private and university hospitals, different countries and cultures, and hospital sizes.

### Funding

The study is not supported by any major grant, but a fundraising campaign among involved universities both in Europe and Latin America will provide the financial means to cover the costs, including insurance where needed and the logistics/human resources of the DQT.

## DISCUSSION

ANDROMEDA-SHOCK-2 is a relevant study in septic shock for several reasons: it proposes a resuscitation strategy based on clinical phenotypes and CRT as a resuscitation target, allowing us to individualize therapy according to the patients’ clinical scenario; it will assess its impact on clinically relevant outcomes; and it will be conducted in a broad range of ICUs on approximately 4 continents, capturing different realities and expanding its external applicability. If our hypothesis proves to be correct, the processes of care of septic shock resuscitation can be optimized with bedside tools.

**Ethics approval and consent to participate:** Each investigator center will submit the study protocol to its Institutional Review Board (IRB). The study will start only after being approved by the IRB. Written informed consent will be obtained from a legal representative of all participants. This study will be performed in compliance with local and international declarations.

**Authors’ contributions:** All the authors helped in the design and the final manuscript draft. All authors read and approved this final manuscript.

## ANDROMEDA-SHOCK-2 INVESTIGATORS:

**Steering Committee:** Glenn Hernández (chair), Jan Bakker (chair), Gustavo Ospina-Tascón (chair), Daniel de Backer, Antoine Vieillard-Baron, Jean-Louis Teboul, Alexandre Biasi Cavalcanti, Eduardo Kattan.

**Advisory Committee**: Elie Azoulay, Maurizio Cecconi, Elisa Estenssoro, Lucas Petri Damiani, Arnaldo Dubin, Olfa Hamzaoui, Fernando Ramasco, Ricardo Castro, Luis Gorodoro.

**Study Coordinating Center**: Olfa Hamzaoui, Fernando Ramasco, Glenn Hernández, Luis GorodoroDelsol, Mario Pozo, Juan Nicolas Medel, Eduardo Kattan, Leyla Alegría, Sebastián Morales.

**Data Safety and Monitoring Board:** Pending.

## STUDY CENTERS:

**Italy:** Humanitas Research Hospital: Maurizio Cecconi, Antonio Messina, Elena Constantini, Fabrio Piccirillo, Alessandro Santini, Massimo Vanoni, Giacomo Iapichino. *Policlinico Paolo Giaccone*: Andrea Cortegiani, Mariachiara Ippolito, Giulia Catalisano, Giulia Ingoglia. *Azienda Ospedaliera Universitaria Policlinico Vittorio Emanuele*: Filippo SanFilippo, Marinella Astuto, Luigi La Via, Veronica Dezio, Bruno Lanzafame, Simone Messina, Eleonora Tringali, Cesare Cassisi, Maria Rita Valenti, Francesco Perna. **Spain:** *Hospital Universitario y Politécnico La Fe*: Paula Carmona García, Iratxe Zarragoikoetxea Jauregui, Marta Lopez Cantero, Miguel Angel Rodenas, Ignacio Albero, Azucena Pajares, José García Canto, Clara Pascual, Victoria Johannesen, Daniel Pérez Ajami, Pedro Martínez Pérez. *Hospital Universitario de León*: Rafael Gonzalez de Castro. *Hospital General Universitario Gregorio Marañón*: Patricia Piñeiro. Hospital Clínic de Barcelona: Adrian Tellez. Hospital Universitario Puerta del Mar: Francisco Miralles Aguiar, Rafael Garcia Hernandez. *Hospital Universitario de La Princesa:* Fernando Ramasco. *Hospital Clínic Universitari de València*: Gerardo Aguilar. *Hospital Universitario de Cruces*: Gonzalo Tamayo. *Hospital Universitario Central de Asturias*: Cristina Iglesias. *Hospital Universitario de Cáceres*: Fernando Montoto. *Hospital General Universitario de Albacete*: Jose Maria Jimenez Vizuete. *Hospital Clínico Universitario Virgen de la Arrixaca*: Carlos Garcia Palenciano. *Hospital Clínico Universitario de Valladolid*: Jose Ignacio Gomez Herreras. *Hospital Universitario Río Hortega*: Cesar Aldecoa. *Complexo Hospitalario Universitario de Ourense*: Conchi Alonso. *Hospital General Universitario de Valencia*: Carolina Ferrer. *Hospital Universitario Marqués de Valdecilla*: Emilio Maseda. *Hospital Povisa*: Sonsoles Leal Ruiloba. *Hospital Meixoeiro*: Elena Vilas. *Hospital Universitario Puerta de Hierro*: Reyes Iranzo. *Hospital General Universitario de Alicante*: Maria Galiana. *Hospital Clínico Universitario de Santiago*: Valentin Caruezo. *Hospital Universitario 12 de Octubre*: Eloisa Lopez. *Hospital Universitario Ramón y Cajal*: David Pestaña. *Hospital Universitario Nuestra Señora de Candelaria*: David Dominguez. *Hospital Universitario de Gran Canaria Doctor Negrín*: Oto Padron. *Hospital General Universitario de Elche*: Ana Pérez. *Hospital del Mar*: Adela Benítez-Cano, Ramón Adalia Bartolomé, Lorena Román Rosa, Mireia Chanzá Albert, Isabel Ramos Delgado. *Hospital Universitario Vall d’Hebron*: Mirian de Nadal. *Hospital Universitario de Mostoles*: Raquel Fernandez. *Hospital de Pontevedra*: Marina Varela. *Hospital Can Misses*: Gaspar Tuero. *Hospital Universitario de Girona*: Marc Vives, Fina Parramon, Maria Diaz, Guillem Pla, Judit Lopez, Nadejda Cuznetova, Neus Sargatal, Berta Baca, Ana Ricart, Iñaki Gascón, Eva Díaz, Cristina Rodríguez. **France:** *Hôpital Bicêtre*: Jean-Louis Teboul. *Hôpital Antoine-Béclère*: Olfa Hamzaoui. *Hôpital Ambroise-Paré*: Antoine VieillardBaron, Cyril Charron, Matthieu Petit. *Hôpital SaintAntoine*: Hafid Ait-Oufella, Jeremie Joffre. *Hôpital Saint Louis*: Elie Azoulay. Hôpital Raymond-Poincaré: Djillali Annane. Hôpitaux Universitaires Henri Mondor: Armand Mekontso Dessap. *Hospices Civils de Lyon*: Matthias JacquetLagrèze, Martin Ruste, Arnaud Ferraris, Delphine Chesnel. *Centre Hospitalier Universitaire de Nancy*: Philippe Guerci. *Centre Hospitalier Universitaire de Nancy*: Mathieu Jozwiak, Jean Dellamonica, Denis Doyen. **United Kingdom:** *King’s College Hospital*: Sam Hutchings, Adrian Wong. **Germany:** *University Hospital Düsseldorf:* Christian Jung. **Belgium:** *Intensive Care Department of the CHIREC Hospitals:* Daniel de Backer. **Netherlands:** *Erasmus Medical Center:* Alexandre Lima. **United Arab Emirates:** *Cleveland Clinic Abu Dhabi:* Jihad Mallat, Fadi Hamed, Baraa Abduljawad, Bruno de Oliveira, Nahla Aljaberi, Yeldho Varghese, Dnyaseshwar Munde, Mohammad Helal, Mohamed Asklaleny, Khaled Ismail. **Israel:** *Rambam Health Care Campus:* Michael Roimi, Danny Epstein, Yaron Bar-Lavie, Roy Ilan. **India**: *Fortis Escorts Hospital:* Supradip Ghosh. **Canada:** *Hôpital Santa Cabrini:* Philippe Rola. University of Manitoba: Asher Mendelson. **United States of America:** *New York University:* Jan Bakker. Cleveland Clinic: Matthew Siuba. *University of Pennsylvania:* John C. Greenwood, Cameron Baston. *Columbia University:* Vivek Moitra. *Wake Forest School of Medicine:* Ashish K Khanna.

**Latin American Intensive Care Network (LIVEN): Mexico:** *Hospital Juárez de México:* Luis Gorordo-Delsol, Jessica Garduño-López, Marcos Amezcua-Gutiérrez, Guillermo Hernández-López. *Instituto Nacional de Ciencias Médicas y Nutrición Salvador Zubirán:* Thierry HernándezGisoul, José Vidal Mayo, Nielzer Rodríguez-Almendros, Gerardo Mercado-Leal, Adrián Valdespino-Trejo, Gabriela Pérez-de los Reyes, Rosario Hernández-Ortega, Fabiola López-Cruz, Eduardo Ríos-Argaiz. *Hospital Civil Fray Antonio Alcalde:* Miguel Ibarra-Estrada, Guadalupe AguirreÁvalos, Diego Jiménez-Pérez, Carlos Gómez-Partida. *Hospital General San Juan del Río:* Orlando Pérez-Nieto, Ernesto Deloya-Tomas, Jorge Carrión-Moya, Jorge LópezFermín, Carlos Mendiola-Villalobos, Gabriela BautistaAguilar. *Hospital H+ Querétaro:* Job Rodríguez-Guillén, Manuel Díaz-Carrillo, Lizzeth Torres-López. *Hospital de Especialidades N° 14 IMSS:* Jesus Salvador Sanchez-Diaz, Carla Gabriela Peniche-Moguel, Maria Verónica CalyecaSanchez, Gustavo Martinez-Mier. **Brazil:** *HCor-Hospital do Coração:* Alexandre Biasi Cavalcanti, Marcela Lopes, Leticia Galvao Barbante, Eliana Vieira Santucci, Erica Regina Ribeiro Sady, Lucas Martins de Lima. *BP - A Beneficência Portuguesa de São Paulo:* Viviane Cordeiro Veiga. **Ecuador:** *Hospital San Francisco de Quito:* Vladimir Granda. *Hospital General IESS Santo Domingo:* Javier Casas Rodriguez. *Hospital IESS Ibarra:* Pedro Torres Cabezas. **Colombia:** *Hospital Universitario Fundación Valle del Lili:* Gustavo Adolfo Ospina-Tascón, Gustavo Andres Garcia Gallardo, Nicolás Orozco Echeverri. *Hospital Santa Clara:* Guillermo Ortiz, Carlos Celemin, Manuel Garay. *Clínica Universitaria Bolivariana:* Francisco Molina Saldarriaga, Álvaro Ochoa, Carlos Blandón, Daniel Rodas, Stella Navarro, Andrés Rivera. *Clínica Las Américas AUNA:* Bladimir Gil Valencia, Rodrigo Murillo Arboleda, David Yepes, Ricardo Orozco, Esteban Aragon, Mario Izaquita. *Hospital Universitario Departamental de Nariño:* Héctor Sánchez Galvez, Álvaro Portilla Cabrera, Mercedes Solarte, Pablo Córdova. *Clínica IPS Universitaria León XIII - Universidad De Antioquia:* Horacio Atehortúa, Diego Patiño, Andrés Hernández, Luis Felipe Atehortua, Oscar Ramos. *Clinica Universidad La Sabana:* Luis Felipe Reyes, Eder Leonardo Cáceres, Katherine Carvajal, Christian Serrano, Yuli Viviana Fuentes, Ana María Crispin. *Hospital Universitario Fundación Santa Fe de Bogotá:* Carlos Santacruz, Amanda Quintairos. *Fundación Clínica Shaio:* Claudia Marcela Poveda, Julián Casallas, Michel Pérez, Lina Saucedo, Ricardo Buitrago. *Hospital Pablo Tobon Uribe:* Nelson Giraldo Ramírez, Gisela de la Rosa Chávez, Carlos Cadavid Gutiérrez, Alex García. **Argentina:** *Hospital Provincial del Centenario:* Juan Carlos Pendino, Lisandro Bettini, Valentín Torres, Juan Ibarzabal. *Clínica La Pequeña Familia:* Fabio German Repetto, David Maria Banegas Litardo, Rocio Castor, Gisela Gómez, Belen Tiseyra, Lucas Flores, Irupe Ramirez, María Luz Campassi. *Sanatorio Otamendi:* Arnaldo Dubín, Vanina Kanoore Edul. *Clínica Bazterrica:* Fernando Pálizas, Bernardo Lattanzio. *Sanatorio Parque:* Cecilia Gonzalez, Jesica Rodriguez Louzan, Santiago Calabrono, Giuliana Sterzer, Luisina Finos, Antonella Lopipi. *Hospital Británico:* Mario Pozo, Gastón Murias, Facundo Gutierrez, Santiago Sac. Hospital CEMIC: Martín Hunter, Ignacio Bonelli, María Fernanda Lurbet. **Uruguay:** *Hospital Español:* Nicolás Nin, Jordan Tenzi, Carlos Quiroga, Pablo Lacuesta, Javier Hurtado. **Chile:** *Pontificia Universidad Católica de Chile:* Glenn Hernández, Eduardo Kattan, Sebastián Morales, Ricardo Castro, Eduardo Espíndola, Leyla Alegria, Vanessa Oviedo, Emilio Daniel Valenzuela Espinoza, Roberto Contreras, Sebastián Bravo, Karla Ramos, Javier Ramírez, Maximiliano Rovegno, Patricio Vásquez, Pablo Born, Magdalena Vera, Rodrigo Ulloa. *Hospital Clínico Regional de Concepción Dr. Guillermo Grant Benavente:* Nicolás Pavez Paredes, Paula Fernández Andrade, Marcos Hernández, Daniela Ponce Holgado, Fabrizio Fasce Villaseñor, Barbara Nahuelpan. *Hospital San Juan de Dios:* David Gallardo, Juan Eduardo Sanchez, José Miguel Arancibia, Alex Muñoz Morales, Florencia Aravena Ibañez, Nelson Lobo Villarroel, German Ramírez Machuca, Gonzalo Galván Escobar, Javier Rojas Vargas. *Hospital Barros Luco Trudeau:* Ronald Pairumani, Carla Araya Armijo, Edward Petruska, Cesar Santis. *Hospital Hernan Henriquez Aravena:* Leandro Ortega, Valentina Toledo. *Complejo Asistencial Dr. Víctor Ríos Ruiz:* Monica Silva Pantoja, Fernando Tirapegui Sanhueza. *Hospital Clínico de la Universidad de Chile:* Juan Nicolás Medel, Rodrigo Cornejo, Jorge Montoya, Nicolás Carreño, César Cortés.
